# Mapping motor point response areas in the calf during transcutaneous neuromuscular electrical stimulation

**DOI:** 10.1186/s12984-026-01999-4

**Published:** 2026-04-25

**Authors:** Nelida Aliaga, Jafeth Lizana, Liv Nilsson Forsström, Robin Juthberg, Paul W. Ackermann

**Affiliations:** 1 Department of Molecular Medicine and Surgery, Karolinska Institutet, Stockholm, Sweden; 2 Department of Neuroradiology, Karolinska University Hospital, Stockholm, Sweden; 3 Department of Trauma, Acute Surgery and Orthopaedics, Karolinska University Hospital, 171 76 Stockholm, Sweden

**Keywords:** Neuromuscular electrical stimulation, Motor point, Electrode placement, Calf muscle, Plantar flexion

## Abstract

**Background:**

Neuromuscular electrical stimulation (NMES) delivered transcutaneously activates skeletal muscle; its effectiveness is determined by stimulation parameters and by how skin-applied currents recruit underlying neuromuscular structures. Motor points (MPs) are commonly used to guide electrode placement; however, they are typically treated as discrete skin locations, and the spatial extent and reproducibility of surrounding responsive regions remain poorly characterised. Moreover, the minimum intensity required to elicit visible motor responses, and its variability over time, are seldom reported. The calf represents an appropriate model to examine these issues, as posterior calf stimulation produces visible ankle plantar flexion (PF). This study aimed to map stimulation response patterns in the skin associated with an identified MP in the posterior calf, assess their reproducibility, and determine the stimulation intensity required to elicit PF.

**Methods:**

Thirty healthy adults underwent three weekly NMES sessions on the same calf. In each session, the same MP was stimulated at the lowest PF-eliciting intensity. A circular grid (5 cm radius) classified the surrounding skin as eliciting PF with muscle contraction (Zone 1), isolated muscle contraction (Zone 2), or no response (Zone 3). Areas were compared across sessions. Temporal stability was assessed using non-parametric tests and bootstrapping. Logistic regression examined associations between participant characteristics and response area size.

**Results:**

A concentric pattern of diminishing responses from the MP was observed. Median Zone 1 (*MP response area*) was 8.1 cm^2^ (Interquartile range (IQR): 4.7–12.6), Zone 2 was 30.4 cm^2^ (IQR: 15.7–44.8), and combined Zone 1 + 2 (*functional MP response area*) measured 42.9 cm^2^ (IQR: 21.5–57.6). Zone 3 was 35.6 cm^2^. All zones showed consistent size across sessions. Higher physical activity and older age were inversely associated with Zone 2 size.

**Conclusion:**

MP activation during transcutaneous NMES is better described as a finite and reproducible cutaneous response area rather than a single skin location. PF is confined to a small, stable core region, while surrounding areas elicit weaker or absent motor responses. These findings have implications for how MPs are conceptualised and assessed in NMES protocols.

**Supplementary Information:**

The online version contains supplementary material available at 10.1186/s12984-026-01999-4.

## Background

Neuromuscular electrical stimulation (NMES) is widely used to activate skeletal muscle in clinical, experimental and performance settings [1–3]. In transcutaneous NMES, the effectiveness of muscle activation depends not only on stimulation parameters, but also on how stimulation delivered at the skin recruits underlying neuromuscular structures [4]. This interaction is influenced by anatomical variability and electrode-related factors such as contact area and coverage, which may affect the consistency and efficiency of the motor response [5, 6].

The concept of the motor point (MP) was first described by Duchenne de Boulogne in 1855 as the site within the muscle where electrical stimulation elicits the most effective muscle contraction at the lowest stimulation intensity [7, 8]. Anatomically, MPs are associated with the nerve’s entry point into the muscle or regions of neuromuscular junctions [9, 10]. In transcutaneous NMES, however, stimulation is delivered through surface electrodes with a finite contact area [11]. As a result, stimulation may recruit underlying motor structures over a broader cutaneous region rather than from a single discrete location [8]. This suggests that the classical MP definition may not fully reflect the clinical reality of NMES.

Despite widespread use of the MP concept, transcutaneous NMES generally treats the MP as a specific location on the skin [1, 12]. However, it remains unclear whether motor responses are confined to this location or can be elicited from neighbouring areas associated with the same MP. While anatomical MP maps have been reported for both upper and lower limbs [12–14], these describe typical anatomical locations across populations and do not address the spatial extent, shape, or temporal consistency of the responsive cutaneous region. In addition, the minimal stimulation intensity required to elicit such responses, and its variability across sessions and between individuals, is rarely reported in MP identification studies.

The calf region provides a model in which transcutaneous stimulation can be directly linked to a visible joint-level motor response [15]. Stimulation of the posterior calf with the knee extended elicits ankle plantar flexion (PF) that can be readily observed and is predominantly attributable to activation of the gastrocnemius muscle [16]. This distal, directionally specific movement enables a direct link between cutaneous stimulation and PF [15, 17].

The aim of this study was to systematically map stimulation response patterns in the skin associated with an identified MP in the posterior calf, defined by visible ankle PF, and to assess the extent and reproducibility of these responses over time. A secondary aim was to quantify the minimal stimulation intensity required to elicit PF at this MP across three weekly sessions and between participants.

## Materials and methods

### Study design and participants

This was a controlled explorative study conducted between November 2023 and March 2024 at the Department of Trauma and Acute Orthopaedic Surgery, Karolinska University Hospital. Thirty healthy participants (15 men and 15 women), aged 18–60 years, were recruited. Participants were eligible if they were not currently hospitalized and had no known medical conditions influencing vascular or neuromuscular function. Exclusion criteria included pregnancy, epilepsy, the presence of a pacemaker or intracardiac defibrillator, cardiovascular disease, grade ≥ 2 hypertension, cerebrovascular disease, any known lower motor neuron damage, or any condition expected to impair calf blood flow, such as vascular injury, deformity, or localized disease of the calf. Participation was voluntary. Demographic data—including age, sex, height, weight, tobacco use, and physical activity level (PAL) (assessed using the Grimby–Frändin scale [18])—were collected through a standardised questionnaire.

### Study procedure

The participants underwent three test sessions on the same leg, each lasting 30 min and scheduled at 7-day intervals (± 1 day). All sessions for a given participant were conducted by the same examiner to ensure procedural consistency. The participants were randomly assigned in a 1:1 ratio to either the left or right leg.

For the purpose of this study, a motor point (MP) was operationally defined as a functional stimulation site, namely the point on the calf’s skin where the lowest intensity of transcutaneous NMES elicited visible plantar flexion (PF) of the foot. PF was defined as flexion of the ankle joint in the sagittal plane, resulting in a downward movement of the foot along its longitudinal axis, and was first identified by the examiner performing the procedure and independently confirmed by a second experienced examiner. This definition was used to clearly differentiate PF from peroneal reflex responses, which typically produce lateral or outward foot movements. Stimulation-induced responses limited to a visible twitch or contraction of the posterior calf muscles, such as the gastrocnemius or soleus, without accompanying PF, were classified as isolated muscle contractions (MC).

The point electrode was applied by a single examiner throughout all assessments, perpendicular to the skin surface, using light and consistent manual pressure sufficient to ensure stable skin contact, without intentional variation to elicit a response.

NMES was delivered using the Chattanooga Theta device (DJO Nordic, Malmö, Sweden), applying the “Motor Point” program (3 Hz, asymmetrical monophasic rectangular pulses, pulse width ~ 380 µs, constant-current mode; 0–120 mA). The active point electrode was used as the cathode (negative polarity; black cable), while the reference electrode was used as the anode (positive polarity; red cable). This choice was based on the lower activation threshold and greater depolarisation of excitable tissue associated with cathodic stimulation, enabling more precise identification of the MP [19, 20]. Stimulation intensity was gradually increased from zero until the lowest level eliciting visible PF, which was recorded in mA. The pen electrode was used to identify the MP, which was marked at this threshold intensity. The MP identified during the first session was used as the reference and was re-identified at the start of each session to ensure consistency across sessions.

Each session consisted of the following three main steps.

1. Definition of the MP search area on the calf (Fig. 1a).

**Fig. 1:**
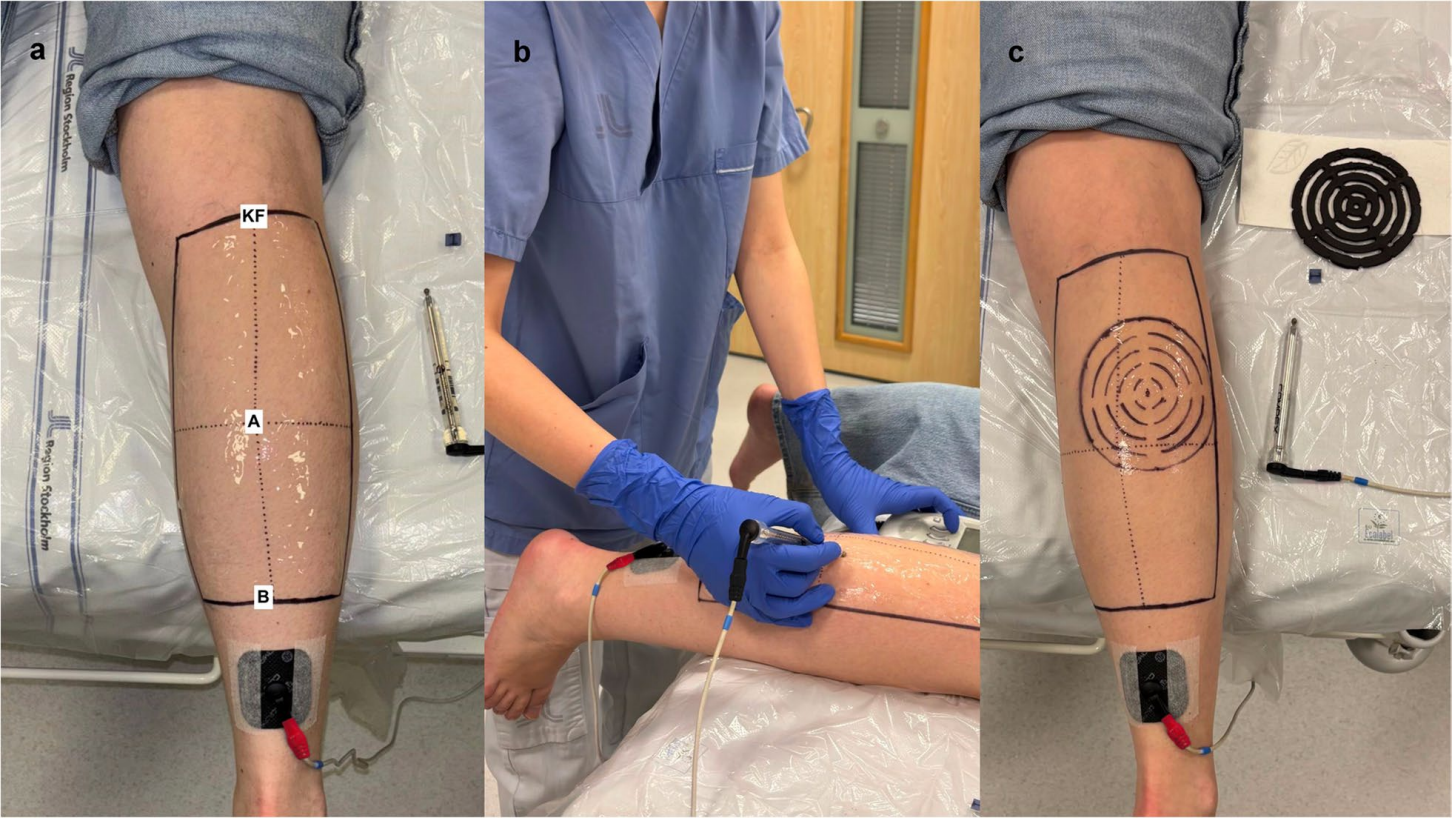
Manual MP search and circular response mapping. **a** The larger rectangular search area, subdivided into four quadrants, was used for manual MP identification; the pen electrode used during the search is visible. **b** Participant’s leg position during the manual MP search. The foot and ankle were suspended freely off the edge of the table, allowing unrestricted ankle movement. The leg was exposed from the knee down. The examiner is performing the manual MP search using a pen electrode connected as the cathode (negative polarity; black cable), with the reference electrode connected as the anode (positive polarity; red cable). **c** After the MP was located, the surrounding skin was systematically scanned at the same NMES intensity used for MP identification, following a circular grid of five concentric circles with 1 cm radial increments. The custom ruler used to trace this grid on the skin is also shown. *MP* motor point, *NMES* neuromuscular electrical stimulation, *KF* knee fold, *A* point A, *B* point B
2. Manual identification of the MP using the pen electrode connected to the NMES device (Fig. 1b).
3. Systematic scanning of a circular area around the MP to evaluate variations in the stimulation response (Fig. 1c).

A detailed description of each procedure is provided below.

### Definition of the MP search area

To define the MP search area, participants lay in a prone position on an examination table with the leg fully exposed and the foot hanging freely over the edge (Fig. 1b). With the knee maintained in an extended position, this set-up ensured that PF occurred against gravity, allowing unrestricted ankle movement and clear visual discrimination between PF and MC responses during stimulation. The centre of the knee fold (KF) was calculated as the midpoint between the medial and lateral tibial condyles. From this point, the posterior midline of the calf (PMC) was defined as a straight line extending to the insertion of the Achilles tendon on the calcaneus.

Two reference points were defined along the PMC:

- Point A, the intersection between the PMC and the maximum circumference of the calf (MCC).
- Point B, located at 60% of the distance from the KF to the Achilles tendon insertion.

The MP search area boundaries were defined as follows (Fig. 1a):

- Proximally, by the circumference at the KF.
- Distally, by the circumference at Point B.
- Medially and laterally, by lines projected from the KF, Point A, and Point B at specific offsets: 15% of the KF circumference at the KF level, and 20% of the calf circumference at both Point A and Point B.

The search area dimensions were guided by anatomical data from cadaveric studies, which locate calf MPs around 40% of lower-leg length measured from the KF (0%) toward the ankle (100%) and within the central ~ 18**–**31% of the mediolateral width[21, 22]. To encompass these regions and account for inter-individual variability, we applied fixed offsets: 20% lateral margins at Points A and B and a 15% proximal margin at the KF. The distal boundary at 60% of lower-leg length was intentionally set distal to the typical cluster of MP locations, establishing practical limits without extending into areas where MPs are uncommon. This layout aligns with a recent in-vivo MP heat-map study[23], which demonstrated high MP probabilities in the central and proximal calf areas, and minimal presence in the lateral or distal regions.

### Manual MP search

The MP search was performed using a pen electrode (DJO Nordic, Malmö, Sweden) connected to the NMES device. The pen electrode featured a small spherical conductive tip (approximately 4 mm in diameter), allowing focal stimulation of localised skin regions. A 5 × 5 cm self-adhesive reference electrode (Compex Snap Performance, DJO Global, USA) was placed 3.5 cm below Point B.

A thin layer of water-soluble conductive gel (Chattanooga TENS-gel) was applied to the MP search area to ensure optimal conductivity. Stimulation intensity was gradually increased in a stepwise manner using the device-specific intensity levels of the Chattanooga system, which range from 0 to 999. Level 0 corresponds to no current output**,** while higher levels correspond nonlinearly to a constant-current output between 0 and 120 mA, depending on pulse width. During testing, stimulation intensity was controlled using these device levels rather than direct milliamperes. For analysis and reporting, device levels were converted to mA according to the manufacturer’s specifications. This conversion was based on the device’s calibration data relating output levels to delivered current under constant-current conditions. All stimulation intensities are expressed in mA. For MP identification, stimulation was applied briefly for localisation purposes and was stopped immediately once visible PF was elicited at the lowest effective intensity. The duration of the MP search was recorded and ranged from approximately 2 to 8 min across participants.

The MP search area was divided into four quadrants, which were scanned sequentially in the following order: superolateral, inferolateral, inferomedial and superomedial (Fig. 1a). The search began at the lowest active device-specific intensity level (level 1), representing the first stimulation level above zero (no stimulation). If no MP was identified within a quadrant, the search continued to the next quadrant using the same intensity. If no MP was found in any quadrant during a complete pass, the stimulation intensity was incrementally increased and the scanning process was repeated until a visible PF response was observed, indicating the localization of an MP.

To enable consistent relocation of the same MP across sessions, the following positional measures were recorded during the first session:

- Distance from the MP to the PMC, measured as the shortest perpendicular distance in centimetres, with the side noted as medial or lateral.
- Distance from the MP to the KF, measured along the limb’s longitudinal axis from the KF transverse level to the MP level, reported in centimetres.

At the start of each subsequent session, the MP was re-identified using the same manual search procedure. Stimulation intensity was then increased stepwise from zero to the minimal effective level that elicited PF at the MP; intensity values from previous sessions were not carried over. The minimal effective intensity was recorded at each session to capture day-to-day variability (e.g., skin–electrode impedance, hydration, recent activity, physiological state).

When two candidate MPs were detected in proximity during the manual search, we first compared their minimal effective intensities. The site requiring the lower intensity was designated MP1 for that participant and used in subsequent sessions. If two sites required the same minimal intensity, MP1 was defined as the site that produced the more distinct PF (greater amplitude and clarity), as agreed by two experienced examiners.

To ensure that neighbouring MPs were truly distinct rather than one broader location, we looked for an inter-MP non-responsive gap—a narrow strip of skin between the two candidates where no visible PF could be elicited when stimulating at the lower of the two minimal intensities (hereafter, “gap”).

### Determining stimulation response variations

After MP identification, stimulation was paused and the circular grid was drawn on the skin using the custom ruler, with the identified MP as the centre. All tests were performed at the same minimal effective intensity that had elicited visible PF during MP identification. The same stimulation program used during MP identification was applied continuously during response mapping while the pen electrode was moved across the skin. Responses at each sampled location were classified as: PF (the MP response area), muscle contraction without ankle movement (MC-only), or no visible response.

**Fig. 2:**
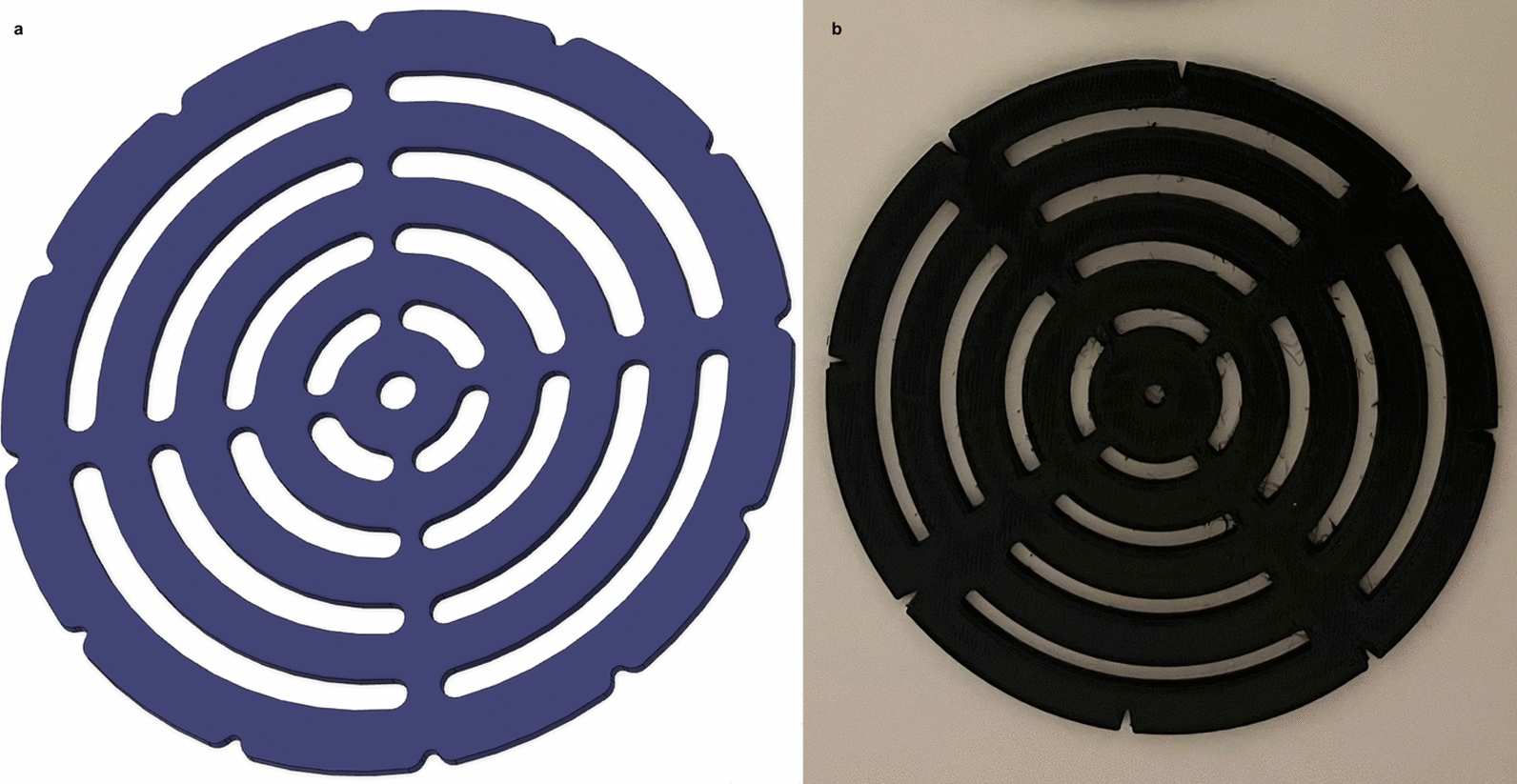
Circular ruler used for mapping stimulation responses. **a** Digital rendering showing concentric circles and angular cutouts. **b** Physical version fabricated via 3D printing

To facilitate systematic and reproducible measurements, a circular ruler was used. It was designed using Autodesk Fusion 360, fabricated from silicone and carbon fibre, and 3D printed with an Ultimaker 2 + at the Karolinska Institutet Innovation Working Lab (Fig. 2).

The choice of a circular design was not intended to imply a circular shape of the MP response area. Rather, it provided a practical and consistent framework for measuring responses in all directions around the MP. This approach minimised potential bias in area sampling and ensured that the response distribution could be objectively analysed. A maximum radius of 5 cm was selected as a predefined methodological boundary based on piloting of the mapping procedure, which indicated that this radius was sufficient to capture spatially localised responses extending away from the MP without assuming a circular distribution, while maintaining a feasible testing duration and limiting participant burden. The ruler incorporated five concentric circles, with radii increasing in 1 cm increments from the MP, combined with angular segmentation at 30° intervals. Although the guide was circular, boundaries were traced as observed; responsive regions were not assumed to be circular**.** This grid allowed for precise documentation of both complete and partial responses within each segment, balancing measurement objectivity with practical usability.

**Fig. 3:**
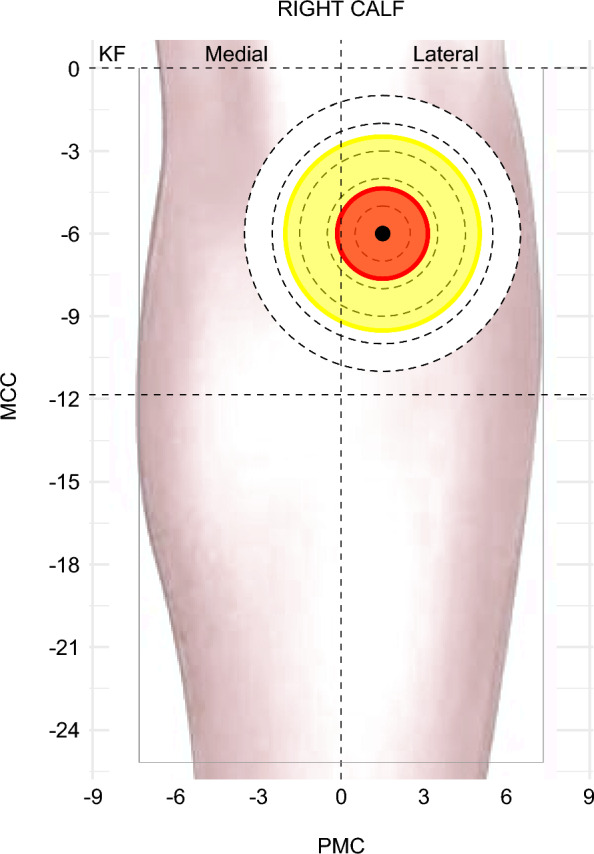
Schematic representation of response zones around an identified MP. The MP, shown as a black dot, was determined at a specific stimulation intensity. Using the same current intensity, the surrounding skin was systematically explored in 1 cm radial increments (five concentric circles shown here for clarity). Colours indicate categorized responses: red = Zone 1 (PF), yellow = Zone 2 (isolated MC without ankle movement), white = Zone 3 (no response). Concentric circles are shown here only to illustrate the mapping procedure, while actual response areas were irregular and quantified as described in the Methods. *MP* motor point, *PF* plantar flexion, *MC* muscle contraction, *KF* knee fold, *PMC* posterior midline of the calf, *MCC* maximum circumference of the calf

The ruler was placed with the MP at its centre, and its edge and cutouts were traced with a marker to create a circular grid around the MP. The NMES device and pen electrode were then used to scan the area using the stimulation intensity at which the MP was initially identified. Scanning began at the innermost circle and proceeded concentrically outwards, with each circumference systematically explored in a clockwise direction, with a maximum of two revolutions per circle. This process was repeated until all five circles were examined. To limit potential fatigue, the mapping protocol included a pause between MP identification and response mapping, a low stimulation frequency (3 Hz), and a limited number of scanning passes. During the scanning of each circle, the examiner recorded variations in the stimulation response, which were classified into three categories (Fig. 3):

- **Zone 1**: PF present. Skin areas in which stimulation elicited visible PF—necessarily reflecting a sufficiently strong muscle contraction—were designated Zone 1.
- **Zone 2**: MC without PF. This category included visible twitches or contractions of the calf muscles that did not elicit a visible PF response. In practice, weaker MCs may not produce PF, and skin areas where such responses were generated were classified as Zone 2.
- **Zone 3**: No visible response. Skin areas within the mapped circular grid where stimulation at the same minimal effective intensity elicited neither PF nor MC, regardless of distance from the MP.

**Fig. 4:**
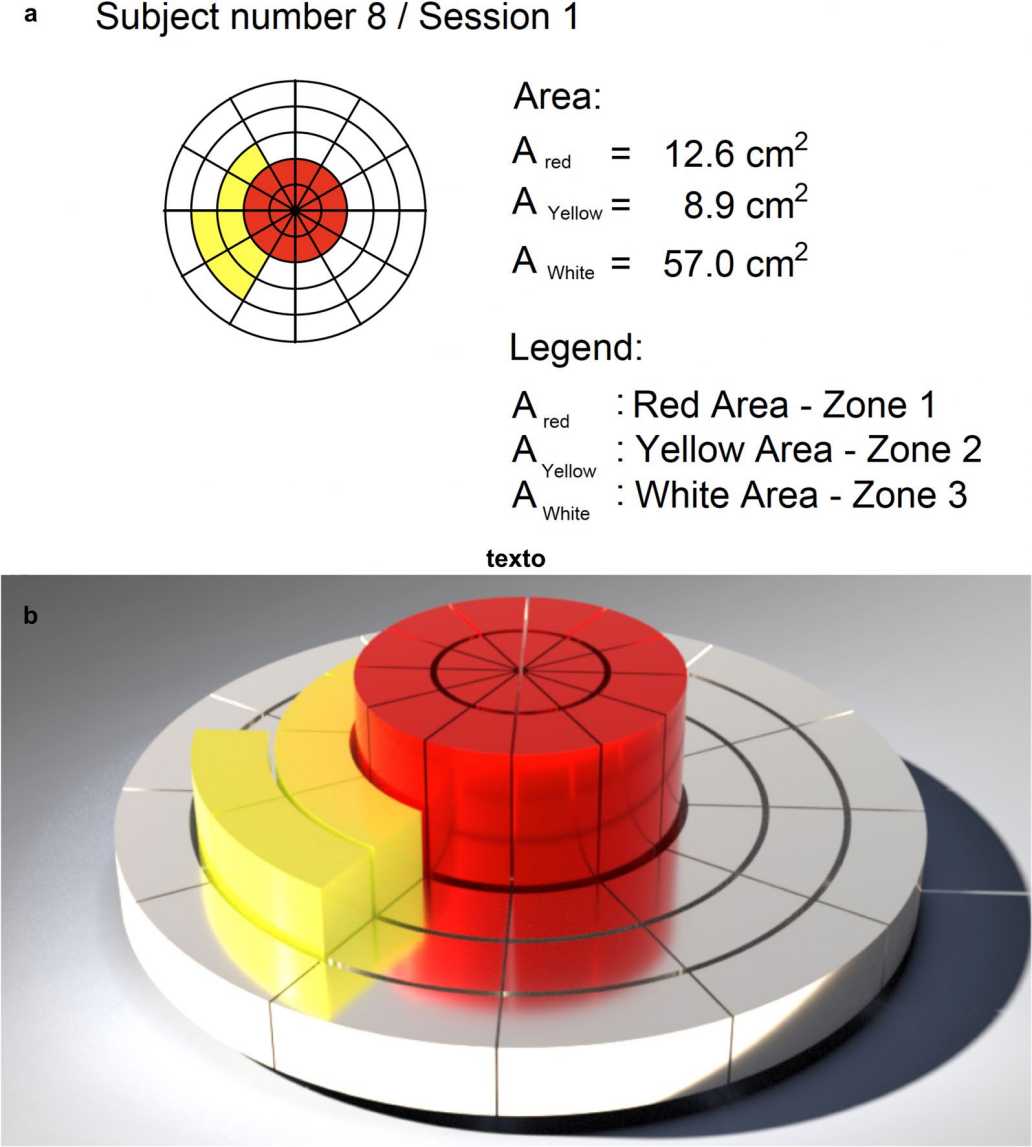
Response zone mapping for a single participant. The figure illustrates one example of the distribution of stimulation responses within the circular search grid around the MP. Red indicates Zone 1 (PF), yellow indicates Zone 2 (MC only), and white indicates Zone 3 (no response). **a** Calculated response areas determined using Autodesk AutoCAD 2025. **b** Three-dimensional graphical representation of the same response zones created with SOLIDWORKS 3D CAD. MP = motor point; PF = plantar flexion; MC = muscle contraction

The responses observed in each segment were documented using a color-coded diagram (red = Zone 1, yellow = Zone 2, white = Zone 3) that mirrored the segmented layout of the search area (Fig. 4). The area corresponding to each response zone was then calculated using Autodesk AutoCAD 2025.

### Statistics

All statistical analyses and visualisations were performed using R software (version 4.3.1). Descriptive statistics summarized participant characteristics. Continuous variables are presented as mean with 95% confidence intervals (CIs) or median with interquartile range (IQR), according to distribution; categorical variables as frequencies and percentages. Normality was assessed using the Shapiro–Wilk test. To evaluate temporal stability across the three sessions, non-parametric Friedman tests were applied, with permutation tests used as a complementary resampling approach. Between-group comparisons (e.g., sex, PAL) were performed using Mann–Whitney U tests and Kruskal–Wallis tests. A two-sided significance threshold of 0.05 was applied. Analyses of Zone 2 and combined Zone 1 + 2 were restricted to participants with a non-zero Zone 2, while Zone 1 was present in all participants.

To obtain robust estimates and quantify uncertainty in nonparametric distributions, non-parametric bootstrapping with 1,000 iterations was applied. This generated empirical sampling distributions of the median, from which 95% CIs were derived. Accordingly, medians are reported together with both IQR (to describe sample spread) and bootstrap-derived 95% CIs (to estimate population uncertainty). Equivalent radii were calculated from the median areas (r = √[A/π]) to provide a scale reference without assuming circularity.

Associations between participant characteristics and Zone 2 size were explored using logistic regression. To satisfy independence, only data from Session 1 were analysed. Accordingly, Zone 2 was dichotomised using the Session-1 median (31.2 cm^2^) as the threshold (0 = below median, 1 = at or above median). Univariable models identified potential predictors, and variables with p < 0.20 entered the multivariable model. Model adequacy was evaluated using Akaike (AIC) and Bayesian (BIC) criteria, and multicollinearity by variance inflation factor (VIF < 5 acceptable).Wald tests evaluated significance of coefficients. Associations were reported using regression coefficients (β), odds ratios (OR), 95% CIs, and p-values.

## Results

### Participant characteristics

Thirty participants (15 women, 15 men) completed the study. Median age was 28 years (IQR: 25–44), median body mass index (BMI) was 24.2 kg/m^2^ (IQR: 22.5–26), and median PAL was 3 (IQR: 3–4). Most participants were nontobacco users (93%). Demographics are summarised in Table 1.

**Table 1:** Demographic characteristics of study participants

Variable	n = 30
Age (years), m (IQR)	28 (25–44)
Height (cm), m (IQR)	168 (158–175)
Weight (kg), m (IQR)	65 (58–82)
Physical Activity Level (1–6), m (IQR)	3 (3–4)
BMI ($\mathrm{kg}/{\mathrm{m}}^{2})$, m (IQR)	24.2 (22.5–26)
Leg (left–right)	14–16
Tobacco use (yes–no)	2–28
Sex (female–male)	15–15

*m* median, *IQR* interquartile range, *BMI* body mass index

### Zone 1: Plantar flexion response

**Fig. 5:**
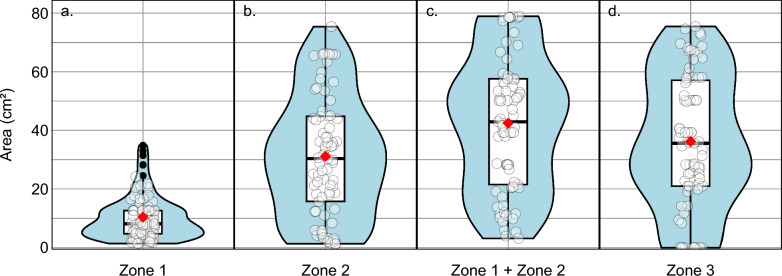
Violin plots showing the distribution of response areas

**Fig. 6:**
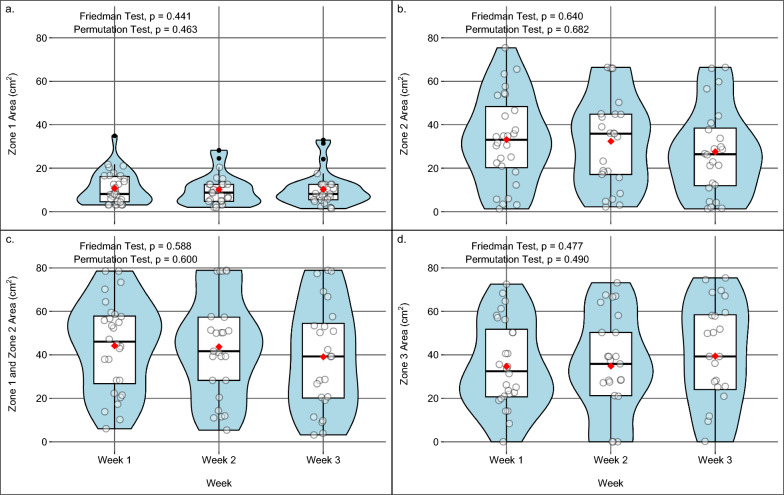
Temporal stability of stimulation response areas across study weeks. Violin plots show the distributions of response area sizes (cm^2^) for Zone 1 (**a**), Zone 2 (**b**), combined Zone 1 + 2 (**c**), and Zone 3 (**d**) measured during Weeks 1–3**.** Boxplots indicate the median (black line) with IQR**,** and red dots represent the mean**.** No statistically significant differences were observed across sessions for any zone**,** indicating high reproducibility of response areas over time (Friedman test with permutation testing; all p > 0.05). *IQR* interquartile range.

Zone 1, defined as the area where stimulation at the lowest effective intensity elicited both MC and visible PF, had a median area of 8.1 cm^2^ (IQR: 4.7–12.6; 95% CI: 7.1–11.0) (Fig. 3, red area; Fig. 5a). This corresponds to a representative circular radius of 1.6 cm (IQR: 1.2–2.0). The size of Zone 1 remained consistent across the three sessions over three weeks (p = 0.441) (Fig. 6a).

Boxplots indicate the median (black line) with IQR, and red dots represent the mean. All areas are expressed in cm^2^. *IQR* interquartile range.

### Zone 2: Muscle contractions without plantar flexion

Zone 2, defined as the skin area eliciting visible MC without PF at the same stimulation intensity, had a median area of 30.4 cm^2^ (IQR: 15.7–44.8; 95% CI: 23.3–35.9) (Fig. 3, yellow area; Fig. 5b). No significant differences were observed across sessions (p = 0.640) (Fig. 6b).

### Combined Zone 1 and Zone 2

The combined responsive area (Zones 1 and 2) had a median of 42.9 cm^2^ (IQR: 21.5–57.6; 95% CI: 39.3–50.9), corresponding to an equivalent circular radius of 3.7 cm (IQR: 2.6–4.3) (Fig. 5c). Weekly analyses showed no significant differences between sessions (Fig. 6c).

### Zone 3: No response

Zone 3, defined as the skin area where stimulation at the same intensity elicited no visible response, had a median area of 35.6 cm^2^ (IQR: 20.9–57.1) (Fig. 5d). No significant differences were observed across sessions (p = 0.477) (Fig. 6d).

### Stimulation intensity across sessions

**Fig. 7:**
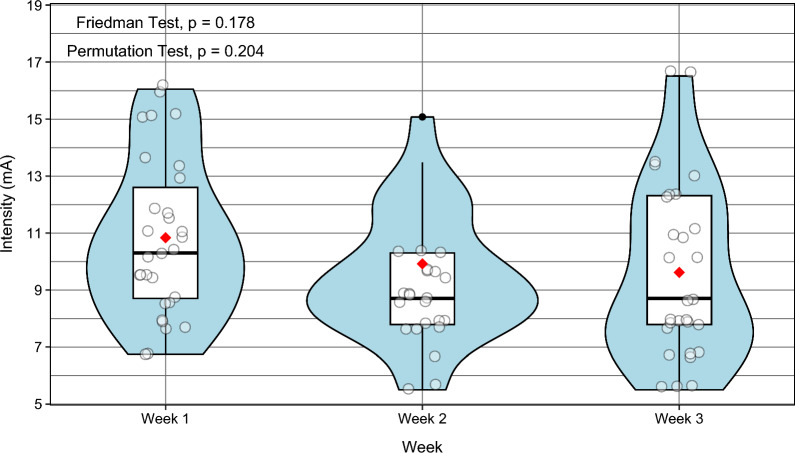
Stimulation intensity required to elicit the response area across study weeks. Violin plots show the distribution of stimulation intensities (mA) during Weeks 1–3. Boxplots indicate the median (black line) with interquartile range (IQR), and red dots represent the mean. Statistical differences across weeks were assessed with Friedman and Permutation tests. *IQR* interquartile range, *mA* milliampere

The intensity required to elicit the defined response areas decreased slightly over time. The median intensities were 10.3 mA (IQR: 8.7–12.6, 95% CI: 9.5–11.7) in week 1; 9.1 mA (IQR: 7.8–11.3, 95% CI: 8.7–10.3) in week 2, and 8.7 mA (IQR: 7.8–12.3, 95% CI: 7.8–11) in week 3. These changes were not statistically significant (p = 0.178) (Fig. 7).

Stimulation intensity (mA) required to elicit visible ankle PF at the same identified MP in each participant across three weekly NMES sessions. Only participants with complete data across all three sessions were included in the analysis; one participant (Subject 6) was excluded, resulting in a final sample of 29 participants, as required for the Friedman test. Values reflect the lowest stimulation intensity at which PF was observed in each session. Participant numbering reflects original study IDs; numbers may appear non-consecutive due to exclusion of participants with incomplete data.

### Multivariable logistic regression

To explore the factors contributing to the observed interindividual variability in Zone 2, multivariable logistic regression was conducted. The final model included age and PAL as explanatory variables. While the model included a statistically significant intercept (p = 0.037), neither age (p = 0.098) nor PAL (p = 0.108) reached statistical significance in the adjusted model.

Supplementary Table 2 summarizes the results of the regression. Both Age and PAL showed an inverse association with the probability of presenting a larger Zone 2. For Age, the odds ratio (OR) was 0.947 (95% CI: 0.9–1.0, p = 0.098), indicating a 5.3% decrease in odds per additional year of age. For PAL, the OR was 0.546 (95% CI: 0.2–1.1, p = 0.108), corresponding to a 45.4% decrease in odds per unit increase in activity level. Although these associations did not reach statistical significance at the conventional 0.05 level, they were explored to identify potential patterns that may warrant further investigation in larger or more targeted cohorts.

## Discussion

This study provides new insights into the responsive skin area surrounding the MP in able-bodied individuals. After identifying the MP at the lowest intensity eliciting PF, the surrounding skin was systematically scanned at that same intensity, allowing responses to be delineated into three zones. Zone 1, where PF (and thus MC) was elicited, may better represent an *MP response area* than a singular anatomical “motor point.”Beyond Zone 1, isolated muscle contractions without PF defined a broader response area—Zone 2—at the same stimulation intensity. Together, Zones 1 and 2 can be considered the *functional MP response area*, as they represent the effective field of NMES activation in practice. Zone 3, by definition, showed no visible response, but its median (~ 35.6 cm^2^) reflects the proportion of skin surrounding the MP where stimulation at this intensity did not elicit a visible motor response. Collectively, these three zones form a concentric-like gradient of decreasing responsiveness from the MP outward.

Zone 1 covered a small, consistent area, while the combined Zone 1 + 2 extended roughly five times larger. Zone 2 alone was more irregular and lacked a representative radius, with its size differing according to individual characteristics. Across sessions, no significant differences were observed in the size of any zone, indicating high reproducibility of these mapped regions over time (Fig. 6). Permutation tests yielded concordant results, and bootstrap analysis further supported this stability by providing consistent confidence intervals around the observed medians. Future integration of quantitative measures of PF–force or joint torque would further enhance standardisation and allow direct comparison of mechanical output across different stimulation sites within the functional MP response area.

Participants fell into two distinct patterns: some displayed only Zone 1 (Zone 2 = 0), whereas others exhibited both zones. In most, Zone 1 exceeded Zone 2, favouring effective PF even with imperfect electrode positioning—a finding that is clinically relevant, as PF with calf contraction produces a stronger physiological muscle pump and greater venous return than MC alone [10, 24]. This observation highlights a functional consequence of effective plantar flexor activation, rather than a treatment-specific application. In a subset, Zone 2 was larger, possibly reflecting current spread or secondary motor entries, further emphasising inter-individual variability in functional motor responses.

The shape and spatial extent of the MP response areas also varied between participants, reflecting differences in muscle fibre orientation, nerve branching, tissue conductivity, and possibly skin-MP distance [21, 22, 25, 26]. Greater distance could attenuate the motor response, although other factors, such as current spread, may allow stimulation to still reach the nerve even from slightly offset regions (Supplementary Fig. 1). From a biophysical perspective, this pattern is consistent with the spatial decay of the electric field in conductive tissues, with a central region reaching the activation threshold of the underlying motor entry point and surrounding regions producing weaker responses [27, 28]. Thus, the mapped response zones likely reflect the cutaneous area from which the MP can be functionally accessed, rather than a spatially distributed MP per se [29]. Accordingly, while schematics (Fig. 3) depict simplified circular areas for clarity, actual boundaries were often irregular. Zone 1 most often appeared as a compact, roughly circular area centred on the MP, consistent with the high repeatability of its boundaries across sessions. This observation further supports the notion that, although the MP is classically defined as a singular anatomical “point,” functional mapping reveals it as a finite surface area—conceptually similar to zooming in on a point and seeing a small circle. However, in some participants Zone 1 appeared elongated or asymmetrical, likely reflecting interindividual anatomical differences. Zone 2, in turn, was typically more irregular and could extend unevenly, possibly due to secondary motor entry points or anisotropic current spread.

This irregularity has practical implications. In scenarios where the MP lies between two electrodes, positioning that captures a substantial portion of adjacent Zone 2 could still contribute to PF through indirect recruitment. These observations reinforce that transcutaneous NMES does not act on a single discrete skin point, but rather within a finite and individually variable cutaneous response area surrounding the MP**.**

From a practical perspective, electrode size has traditionally been discussed in relation to selectivity, comfort, and current density during NMES applications. Prior work has suggested that very small electrodes (< 8 cm^2^) may increase the risk of discomfort or require higher stimulation intensities, whereas very large electrodes (> 43 cm^2^) may reduce selectivity by activating broader muscle regions or multiple motor entry points [5, 30, 31]. However, the present study did not directly compare electrode sizes or configurations and therefore does not support specific electrode size recommendations. Instead, our findings indicate that the cutaneous region capable of eliciting motor responses at a given intensity is finite and individually variable, providing a physiological context for interpreting how electrode dimensions may interact with *functional MP response areas*.

Electrode coverage is also critical for enhancing venous return through gastrocnemius contraction, a cornerstone of thromboprophylaxis in individuals at risk of venous stasis [2, 32]. The present findings demonstrate that motor activation during transcutaneous NMES occurs within a finite and individually variable cutaneous response area surrounding the MP, rather than at a single discrete skin point. In this context, the observed variability in *MP response areas* and *functional MP response areas* provides a physiological basis for understanding how stimulation fields interact with functionally responsive skin regions during NMES. Accordingly, our findings underscore that MP activation during transcutaneous NMES depends on a spatially extended and individual-specific response area, challenging the classical representation of the MP as a single skin location.

Stimulation intensity required to elicit PF at the identified MP showed a small, non-significant decrease across the three sessions (Fig. 7). Although this trend did not reach statistical significance, intensity values within participants were generally consistent across sessions, indicating temporal stability of the plantar-flexion–eliciting threshold at the same MP. In contrast, the absolute stimulation intensity required to elicit PF varied between participants (Supplementary Table 1), highlighting inter-individual differences in neuromuscular activation thresholds. Reporting these values provides important context for the mapped response areas, as the current required to elicit a visible joint-level response reflects the interaction between the stimulation field and underlying neuromuscular structures and can be influenced by individual anatomical and biophysical factors such as sex, tissue composition, fat distribution, and skin impedance. Even modest reductions in the current required to achieve a visible motor response are relevant for comfort and tolerability during repeated NMES use. The observed between-participant variability warrants further investigation in future studies.

Multivariable regression suggested that higher PAL and older age were associated with smaller Zone 2, together explaining approximately 15% of the variance (Supplementary Table 2). Although modest, this variance suggests a physiological contribution of age and activity level to the spatial spread of NMES responses in able-bodied individuals**.** A plausible explanation is that individuals with higher physical activity levels may have greater muscle mass and lower fat mass, which can reduce the distance between the skin and muscle, thereby enhancing the focality of stimulation and limiting the spread of the Zone 2 response. Similarly, age-related changes in muscle composition and skin properties may influence the transmission of electrical stimulation through the tissue. Taken together, these findings indicate that individual characteristics can modulate the spatial extent of *functional MP response areas,* rather than reflecting uniform NMES behaviour across participants. To our knowledge, this is the first study to investigate how individual participant characteristics may modulate the spatial extent of MP response areas during NMES.

## Limitations

This study has some limitations. The sample size included 30 healthy participants, which may limit generalizability to other populations with different body compositions or clinical conditions. The study was conducted under controlled conditions, which may not fully reflect real-world NMES use. The effects of fatigue, skin hydration, or adaptations beyond three sessions were not evaluated. Future studies should incorporate longer follow-up periods and monitor these factors. A further limitation is that the elicited motor response was assessed qualitatively, based on visual confirmation of PF, rather than using quantitative measures of force or joint torque. This limits standardisation of the procedure and prevents direct comparison of the mechanical output produced at different stimulation sites within the mapped response areas. An additional limitation is the use of a Chattanooga device with stepwise increments, which prevented continuous fine-tuning of stimulation intensity. With systems allowing smaller incremental adjustments under ideal conditions (e.g., without fatigue or adaptation), response area boundaries might have been delineated with greater precision. Lastly, the study used a fixed electrode setup and did not examine how variations in electrode size, shape, or configuration influence the interaction with MP response areas. Functional outcomes associated with different electrode configurations were also not assessed.

## Conclusion

This study demonstrates that the MP identified by transcutaneous NMES is best understood not as a single skin location, but as a finite and reproducible cutaneous response area. By systematically mapping stimulation responses at a constant intensity, we show that PF is confined to a small, stable core region, while broader surrounding areas elicit weaker or absent motor responses. The high temporal stability of these response areas across sessions supports their reliability for repeated assessments. Together, these findings provide a physiological framework for understanding functional MP behaviour during NMES and highlight the importance of accounting for inter-individual variability when analysing or designing stimulation-based protocols.

## Supplementary Information

Additional file 1: Supplementary Figure 1. Conceptual schematic illustration of cutaneous MP response areas and underlying motor nerve. The diagram is not an exact anatomical rendering but represents how electrical stimuli traverse skin, subcutaneous tissue, and muscle to reach the motor nerve. (a) Zone 1 (PF, shown in red–pink) denotes the skin region where minimal-intensity stimulation with the pen electrode consistently elicits PF. (b) Zone 2 (MC only, shown in yellow) corresponds to the surrounding area where the same minimal intensity produces only muscle contractions. This reduced output may reflect, in part, a greater skin-to-MP distance, though other factors are likely involved. Movement of the pen electrode within Zone 2 illustrates that the MP belongs to a broader response area rather than a single pinpoint location. Variability in the size and shape of these zones across individuals highlights the spatial relationship between surface stimulation and the underlying MP. Abbreviations: MP = motor point; PF = plantar flexion; MC = muscle contraction.

Additional file 2.

## Data Availability

The datasets generated and analysed during the current study are available from the corresponding author on reasonable request.

## Abbreviations

AIC: Akaike Information Criterion
BIC: Bayesian Information Criterion
BMI: Body mass index
C-NMES: Calf Neuromuscular Electrical Stimulation
CI: Confidence interval
IQR: Interquartile range
KF: Knee fold
MCC: Maximum Calf Circumference
MC: Muscle contraction
MP: Motor point
NMES: Neuromuscular electrical stimulation
OR: Odds ratio
PAL: Physical activity level
PF: Plantar flexion
PMC: Posterior midline of the calf
SE: Standard Error
VIF: Variance inflation factor
