# Supplementary figures and images for: Mapping motor point response areas in the calf during transcutaneous neuromuscular electrical stimulation
Source: J Neuroeng Rehabil. 2026 Apr 25;23:145. doi: 10.1186/s12984-026-01999-4 (PMC13127072; doi:10.1186/s12984-026-01999-4)

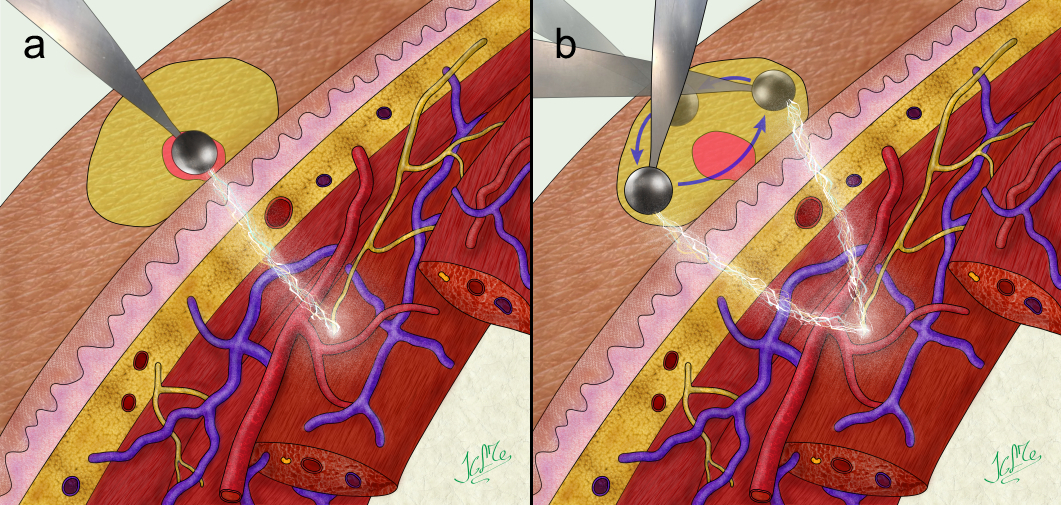

Supplement: Supplementary file 1 — Additional file 1: Supplementary Figure 1. Conceptual schematic illustration of cutaneous MP response areas and underlying motor nerve. The diagram is not an exact anatomical rendering but represents how electrical stimuli traverse skin, subcutaneous tissue, and muscle to reach the motor nerve. (a) Zone 1 (PF, shown in red–pink) denotes the skin region where minimal-intensity stimulation with the pen electrode consistently elicits PF. (b) Zone 2 (MC only, shown in yellow) corresponds to the surrounding area where the same minimal intensity produces only muscle contractions. This reduced output may reflect, in part, a greater skin-to-MP distance, though other factors are likely involved. Movement of the pen electrode within Zone 2 illustrates that the MP belongs to a broader response area rather than a single pinpoint location. Variability in the size and shape of these zones across individuals highlights the spatial relationship between surface stimulation and the underlying MP. Abbreviations: MP = motor point; PF = plantar flexion; MC = muscle contraction. [file 12984_2026_1999_MOESM1_ESM.jpg]
