# Supplementary material for: Mapping motor point response areas in the calf during transcutaneous neuromuscular electrical stimulation
Source: J Neuroeng Rehabil. 2026 Apr 25;23:145. doi: 10.1186/s12984-026-01999-4 (PMC13127072; doi:10.1186/s12984-026-01999-4)
Supplement: Supplementary file 2 — Additional file 2. [file 12984_2026_1999_MOESM2_ESM.docx]

**Supplementary Table 1**: Stimulation Intensity to elicit PF at the identified MP across three weeks

| Subject Number | Intensity (mA) | | |
| --- | --- | --- | --- |
|  | Week 1 Week 2 Week 3 | | |
| 1 | 10.3 | 10.3 | 6.7 |
| 2 | 9.5 | 9.5 | 11.0 |
| 3 | 9.5 | 9.5 | 10.3 |
| 4 | 7.8 | 10.3 | 6.7 |
| 5 | 15.1 | 23.0 | 10.3 |
| 6 | 13.5 | 15.1 | NA |
| 8 | 15.1 | 12.3 | 12.3 |
| 9 | 12.9 | 12.3 | 13.5 |
| 10 | 11.0 | 13.5 | 11.0 |
| 11 | 16.1 | 11.7 | 16.5 |
| 12 | 9.5 | 8.7 | 5.5 |
| 13 | 8.7 | 8.7 | 8.7 |
| 14 | 11.7 | 8.7 | 7.8 |
| 15 | 11.7 | 12.3 | 11.0 |
| 16 | 16.1 | 7.8 | 12.3 |
| 17 | 13.5 | 8.7 | 16.5 |
| 19 | 10.3 | 10.3 | 13.5 |
| 20 | 7.8 | 9.5 | 12.3 |
| 21 | 11.7 | 11.7 | 12.9 |
| 22 | 6.7 | 7.8 | 7.8 |
| 24 | 11.0 | 8.7 | 7.8 |
| 25 | 15.1 | 8.7 | 7.8 |
| 26 | 10.3 | 7.8 | 7.8 |
| 27 | 7.8 | 5.5 | 5.5 |
| 28 | 11.0 | 7.8 | 5.5 |
| 29 | 7.8 | 7.8 | 6.7 |
| 30 | 8.7 | 5.5 | 6.7 |
| 31 | 6.7 | 6.7 | 7.8 |
| 32 | 8.7 | 7.8 | 7.8 |
| 33 | 9.5 | 9.5 | 8.7 |

| **Supplementary Table 2**. Multivariable Logistic Regression Results | | | | | |
| --- | --- | --- | --- | --- | --- |
| Variable | Coefficient (β) (Estimate) | Standard Error (SE) | Odds Ratio (OR) | 95% CI for OR | p - value |
| Intercept | 4.089 | 1.959 | 59.730 | 1.800–4802.035 | 0.037 |
| Age | –0.054 | 0.033 | 0.947 | 0.882–1.006 | 0.098 |
| PAL | –0.606 | 0.377 | 0.546 | 0.236–1.088 | 0.108 |

Multivariable logistic regression results for the probability of presenting a larger Zone 2 (threshold = median, 31.2 cm²). P-values correspond to Wald tests. Abbreviations: β = regression coefficient; SE = standard error; OR = odds ratio; CI = confidence interval; PAL = Physical Activity Level.
